# Jessner’s Solution with Trichloroacetic Acid Chemical Peel: Optimizing Outcomes and Safety

**DOI:** 10.1097/GOX.0000000000002250

**Published:** 2019-05-17

**Authors:** Erez Dayan, Rod J. Rohrich

**Affiliations:** From the Dallas Plastic Surgery Institute, Dallas, Tex.

## Abstract

Trichloroacetic acid (TCA) is a versatile agent, efficacious in treating a spectrum of facial rhytids and dyschrommias at varying concentrations. TCA is commonly used in a 30%–35% concentration to achieve a medium-depth peel into the upper reticular dermis. The addition of Jessner’s solution before the TCA peel application leads to partial removal of epidermis, allowing for deeper penetration of the TCA. This combination is beneficial, as lower concentrations of TCA can be used for the same depth of peel, minimizing complications such as scarring.

## Abstract 2

Supplemental Digital Content is available in the text.

Chemical treatments to enhance facial appearance are among the earliest forms of aesthetic procedures, dating back to ancient Egyptians who used animal oils, salt, alabaster, and sour milk to improve the skin.^1^ Today, a variety of formulations and concentrations are used to treat conditions such as dyschromias, rhytids, actinic changes, and keratosis.^2^ The type of chemical peel is often selected based on depth of penetration required to effectively treat a given condition. As such, chemical peels are frequently classified based on depth of penetration (superficial, medium, and deep) (Table 1).^2^ Light peels resurface the epidermal layer and most commonly include alpha hydroxyl acids such as glycolic acid or Jessner’s solution (resorcinol, salicylic acid, lactic acid, and ethanol). Medium peels include trichloroacetic acid (TCA) 30%–35% and reach the papillary dermis. Deep peels affect the deep papillary dermis and include the Baker Gordon phenol formula peel.^3,4^

**Table 1.**
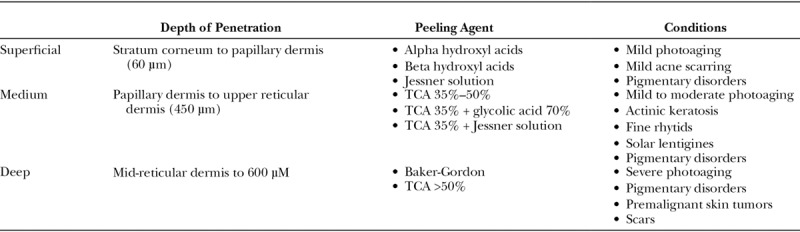
Types of Chemical Peels and Depth of Treatment

With the increasing popularity of laser skin resurfacing over the past 20 years, the art of chemical peeling is often discounted in plastic surgery training. This may lead to suboptimal results, as the clinician must have strong working knowledge on the type of solution, concentration, patient skin type, application technique, peri-procedural care, and repetition of treatment.^5^

## TRICHLOROACETIC ACID AND JESSNER’S SOLUTION

Trichloroacetic acid is a versatile agent, efficacious in treating a spectrum of facial rhytids at varying concentrations. TCA is commonly used in a 30%–35% concentration to achieve a medium-depth peel into the upper reticular dermis.^5^ Importantly, a number of factors other than the concentration of TCA contribute to the depth of peel obtained such as skin preparation, pretreatment skin type, and method of application. The depth of peel can be determined clinically based on the frost color as described by Dolezal (Table 2).^6,7^

**Table 2.**
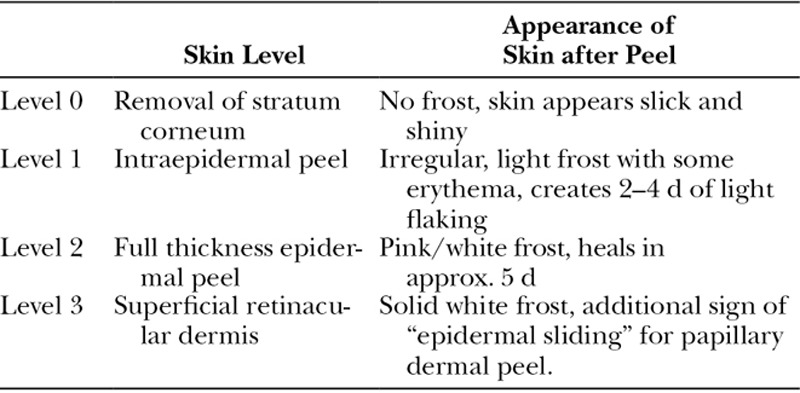
Appearance of Skin for Given Level of Chemical Peel

The addition of Jessner’s solution before TCA peel application leads to partial removal of epidermis, allowing for deeper penetration of the TCA. This combination is beneficial, as lower concentrations of TCA can be used for the same depth of peel, minimizing complications such as scarring.^5^

### Preprocedure Protocol

Consultation with the patient should be used to establish realistic goals and expectation as well as to educate the patient on important perioperative care instructions for optimal results. A careful history and physical examination allow the clinician to determine the patients’ candidacy (Table 3).

**Table 3.**
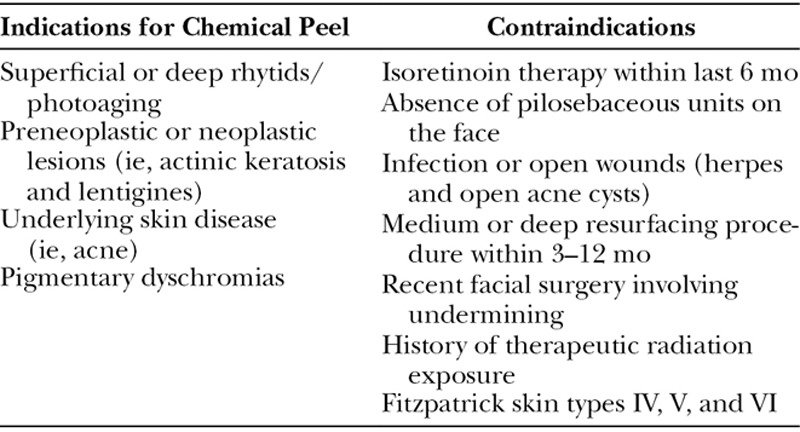
Indications and Contraindications to Chemical Peel

The senior author’s (R.J.R.) preference is to pretreat all patients for 4–6 weeks before chemical peeling.^5,8^ This regimen includes topical tretinoin (0.05%–0.1%), hydroquinone (2%–4%), sunscreen, and alpha hydroxyl acid (4%–10%). Pretreatment improves skin tolerance, regulates fibroblast and melanocyte function, improves dermal circulation, and allows for the treated skin to heal 3–4 days faster due to increased cellular division and new collagen formation.^1,5,9^ Modifications to this preprocedure regimen (dosages and application intervals) are made as needed based on tolerance and skin types. A week before peel, patients are started on a cleansing and toning protocol and encouraged to maintain adequate hydration and moisturize of the skin. Acyclovir is initiated 2 days before chemical peel and continued 5 days after the peel in patients with prior history of herpetic lesions.

### Procedure

Safety and consistency are prioritized to ensure optimal results (**SDC1**) (**see** video, Supplemental Digital Content 1 which displays 30% TCA peel with Jessner’s solution technique, http://links.lww.com/PRSGO/B58). This begins with a setup of 4 clearly labeled glasses ordered from left to right in the appropriate sequence of usage. The glasses are filled by the operating surgeon with: (1) 70% ethyl alcohol, (2) acetone, (3) Jessner’s solution, and (4) 35% TCA acid solution.^5^ In our practice, the Jessner’s solution is premixed by a pharmacist and contains 100 mg of 95% ethanol, 14 g of resorcinol, 14 g of salicylic acid, and 14 ml of lactic acid. Also, available on this table are 2 × 2 gauze and cotton tip applicators.

**Video Graphic 1.**
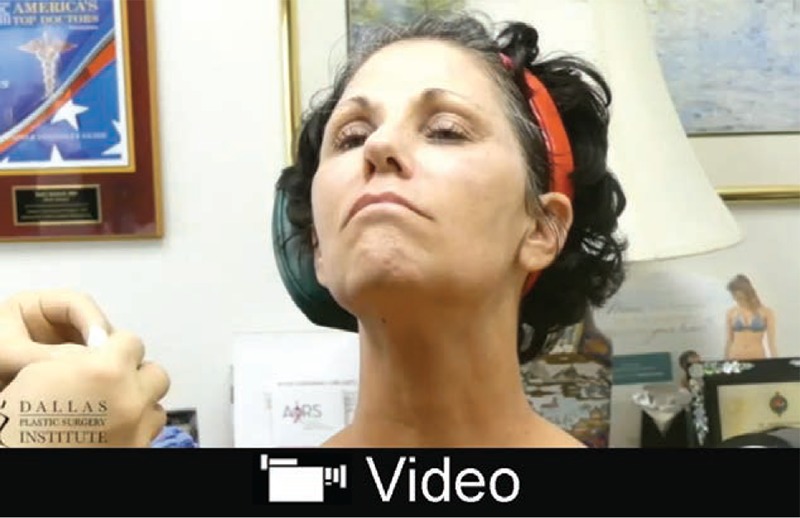
See video, Supplemental Digital Content 1 which displays 30% TCA peel with Jessner’s solution technique. This video is available in the “Related Videos” section of the Full-Text article on PRSGlobalOpen.com or available at http://links.lww.com/PRSGO/B58.

The patient’s skin is anesthetized approximately 40–60 minutes preprocedure with topical anesthetic (Lidocaine/Prilocaine cream). The procedure begins with cleansing of the skin using 2 × 2 gauze soaked in 70% ethyl alcohol. Next, acetone is applied in a similar manner as a degreasing agent. Once this is complete, the skin is allowed to air dry. Jessner’s solution is then applied to exfoliate and peel the stratum corneum of the epidermis. The depth of the Jessner’s peel is controlled by number of applications.^4^ One to four layers of Jessner’s solution is applied to the face with the endpoint being uniform areas of erythema with slight areas of frost.^4,5^ In patients with thick sebaceous skin, more coats of Jessner’s may be required. After the Jessner application is complete, 35% TCA solution is applied. The application of Jessner’s solution and TCA is similar; 2 × 2 gauze is saturated with the solution and wrung out to avoid any dripping. The senior author uses a 3-finger technique to allow for a wide and consistent surface area to be covered.^5^ A cotton tip applicator wrung with TCA is used to treat rhytids in the periorbital and perioral region. The skin in these areas is stretched to allow for the peel to reach the bottom of the rhytids. The wooden end of the cotton tip applicator can be used for selective application of the peel for deeper rhytids.^5^ The margin of the area being peeled (typically mandibular border for facial peel) is lightly feathered to allow for a natural and inconspicuous transition. These areas are all constantly reassessed for color changes to assess depth and efficacy of the peel.

### Postprocedure Care

Once the peel is complete a thin layer of Bactroban ointment is applied to the treated areas. The patients are instructed not to moisturize the area, as this will impede desired sloughing.^5,10^ Typically, 7–10 days are needed for skin to slough and reepithelialize.^1^ Dermal regeneration takes up to 6 weeks. Patient may wash their faces daily without scrubbing for the first 3 days. They are instructed to pat dry the area with a soft towel. Cold compresses and anxiolytics are used to minimize discomfort and oral narcotics may be used as needed. All patients are given 24 hours of prophylactic antibiotics. Once the skin reepithelializes (7–10 days) the aforementioned preprocedure regimen is restarted.^5^

## SUMMARY

In select patients, combination of Jessner’s solution with 35% TCA solution allows for a safe and effective resurfacing of moderate facial rhytids and dyschromias. Pre- and postprocedure skin care in addition to systematic application of the chemical peel optimizes results while minimizing potential complications.

## Supplementary Material
